# Supplementary material for: Probing the biogenesis pathway and dynamics of thylakoid membranes
Source: Nat Commun. 2021 Jun 9;12:3475. doi: 10.1038/s41467-021-23680-1 (PMC8190092; doi:10.1038/s41467-021-23680-1)
Supplement: Supplementary file 1 — Supplementary Information [file 41467_2021_23680_MOESM1_ESM.pdf]

Supplementary Information

For

Probing the biogenesis pathway and dynamics of thylakoid membranes

Huokko et al.

Including:

Supplementary Figures 1-15

Supplementary Table 1

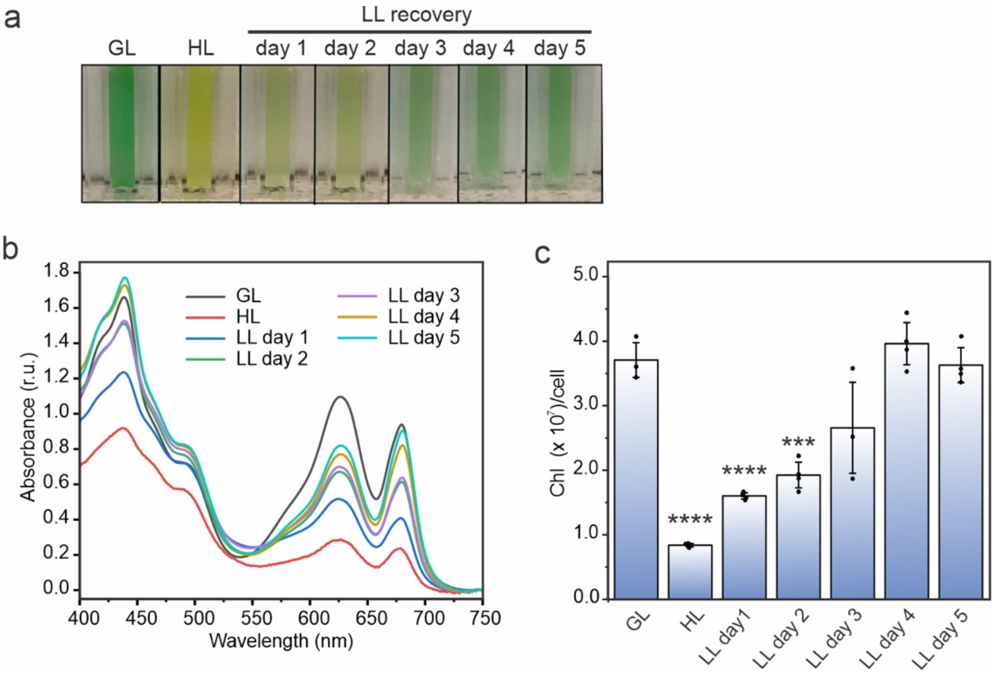

Supplementary Figure 1. The pigment composition during light-regulated thylakoid membrane biogenesis in *Synechococcus*. Cells were grown under growth light (GL), high light (HL) and HL-grown cells were transferred to low light (LL) conditions for 5 days. a, Representative color phenotypes of cells from 3 biologically independent experiments. OD_{750} was adjusted to 0.6 before imaging. **b**, The whole-cell absorption spectra at room temperature. n = 3 biologically independent preparations for GL and LL day 3; n = 4 biologically independent preparations for HL, LL day 1, LL day 2, LL day 4 and LL day 5. Curves were normalized to 750 nm. **c**, The Chl amount (Chl molecules × 10^7 per cell). Values are means ± SD; n = 3 biologically independent preparations for GL and LL day 3, and n = 4 biologically independent preparations for HL, LL day 1, LL day 2, LL day 4 and LL day 5. Asterisks indicate the statistically significant difference compared to GL cells. For HL $p = 1.0 \times 10^{-5}$, for LL day 1 $p = 4.89 \times 10^{-5}$ and for LL day 2 $p = 3.68 \times 10^{-4}$. Statistical analysis was performed using two-sided two-sample t-Test.

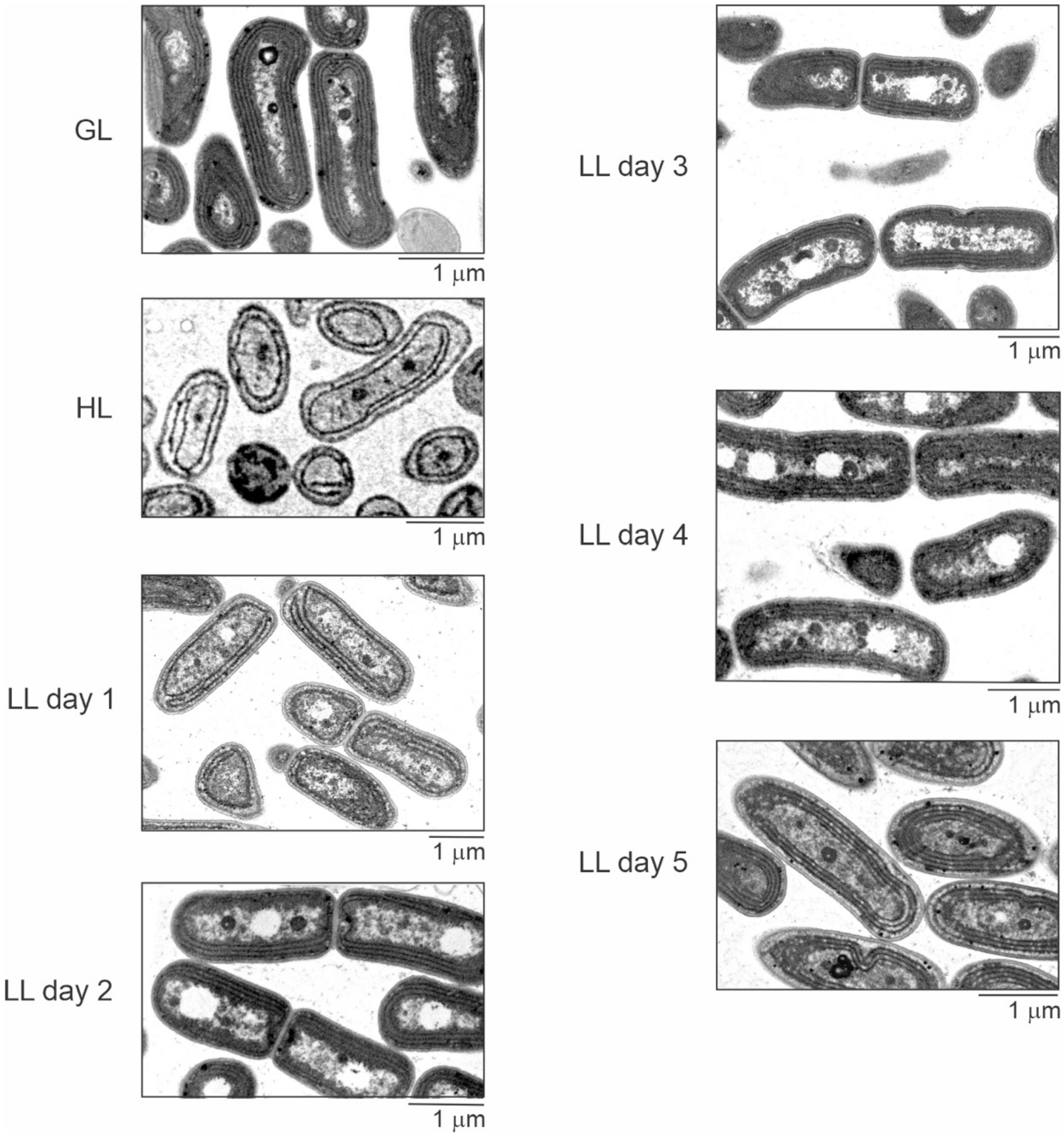

Supplementary Figure 2. Thin-section transmission electron microscopy during light-regulated thylakoid membrane biogenesis in *Synechococcus*. Cells were grown under growth light (GL), high light (HL) and HL-grown cells were transferred to low light (LL) conditions for 5 days. See also Fig. 1a. Representative TEM images were derived from at least three biologically independent preparations with similar results.

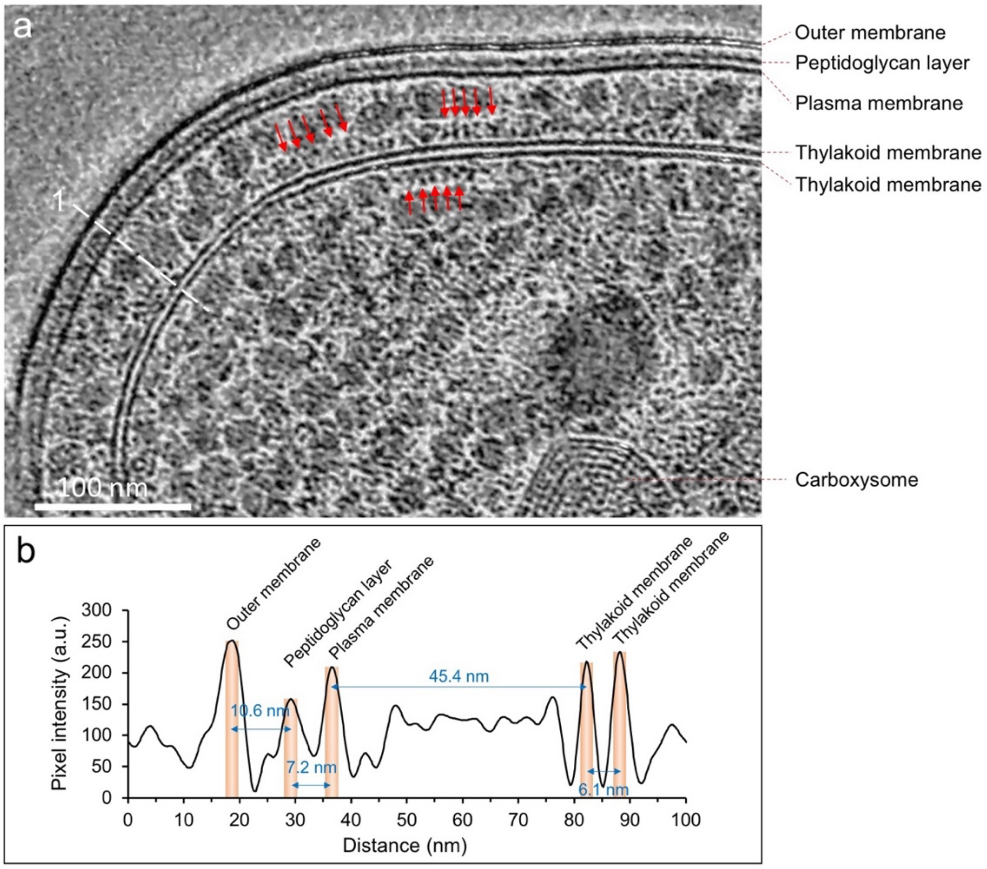

Supplementary Figure 3. Analysis of the cryo-ET of the *Synechococcus* cell grown under HL. a, *in situ* cryo-ET of the *Synechococcus* cell shown in Fig. 2A. Red arrows indicate the phycobilisomes that are densely arranged on the cytoplasmic surface of thylakoid membranes. A line scan (1, line width = 10 pixels) across the outer membrane and thylakoid membranes was used to measure the distances between cellular membranes. **b**, cross-section profile from the line scan indicated in **a**. The outer membrane, peptidoglycan layer, plasma membrane and thylakoid membranes are indicated. a.u., arbitrary units. Profile analysis was performed with Fiji (ImageJ, NIH). The distances between each cellular layer are indicated.

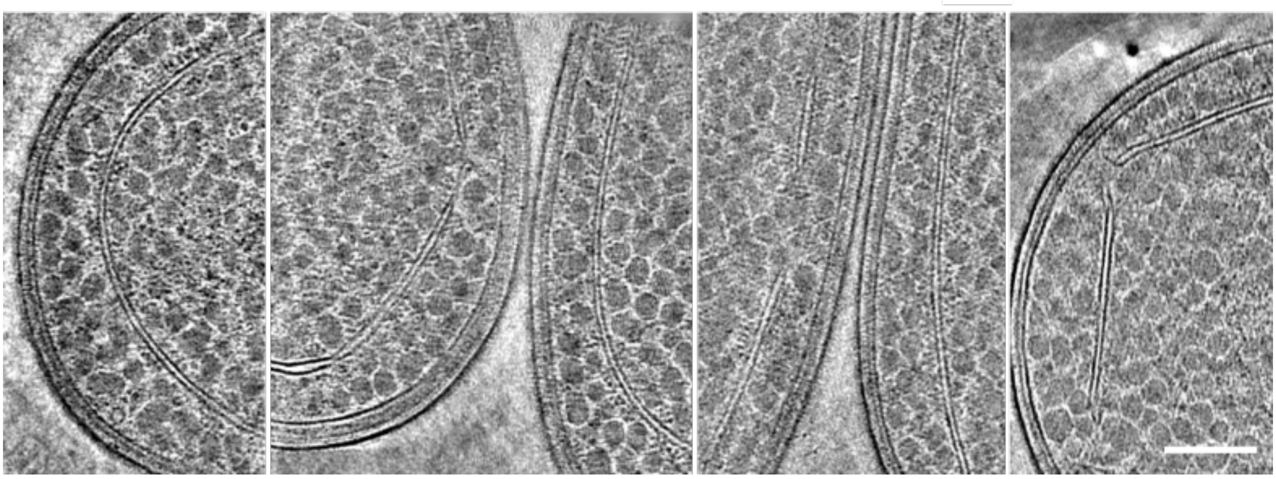

Supplementary Figure 4. **Tomographic slices of representative thylakoid membranes of *Synechococcus*, showing no connections between thylakoid and plasma membranes.** Slice thickness, 2.49 nm. Scale bar, 200 nm. Experiment was repeated 3 times with similar results.

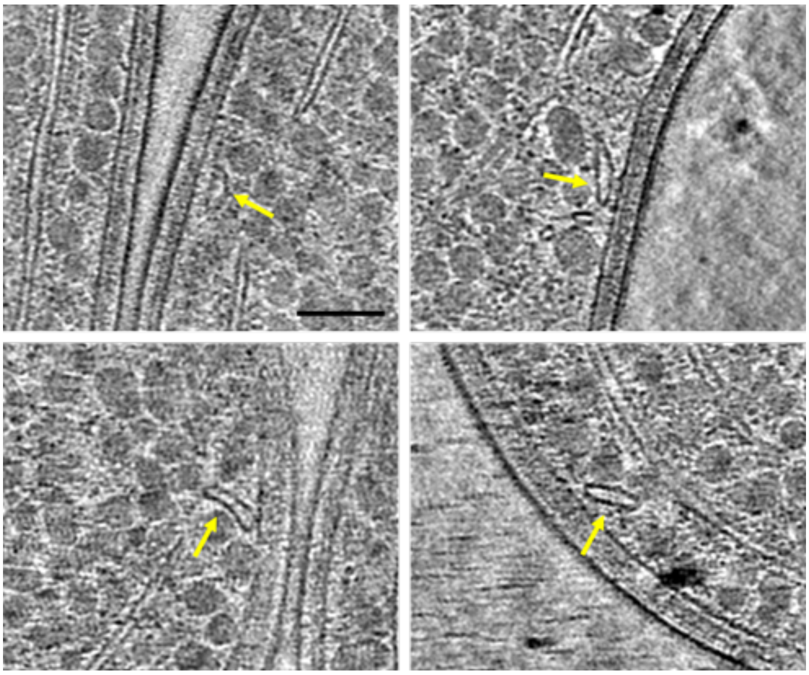

Supplementary Figure 5. A gallery of small segments of thylakoid membranes in *Synechococcus* near the thylakoid membrane breakages close to the plasma membrane (yellow arrows). Slice thickness, 2.49 nm. Scale bar, 100 nm. See Supplementary Movies 3, 4. Experiment was repeated 3 times with similar results.

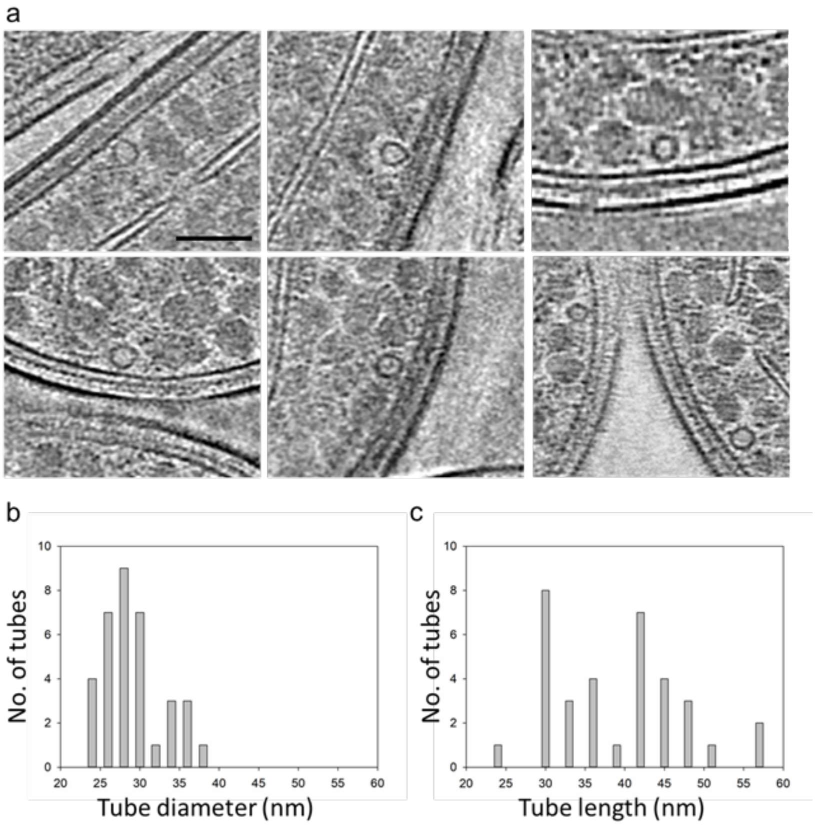

Supplementary Figure 6. **Morphology of the tubular structures *in Synechococcus*. a**, A gallery of short tubular structures located beneath the cell membrane in tomographic reconstructions. Experiment was repeated 3 times with similar results. **b**, Distribution of the diameter of the tubular structures, *n* = 35 tubular structures. **c**, Distribution of the length of the tubular structures, *n* = 35 tubular structures. Slice thickness, 2.49 nm. Scale bar, 100 nm.

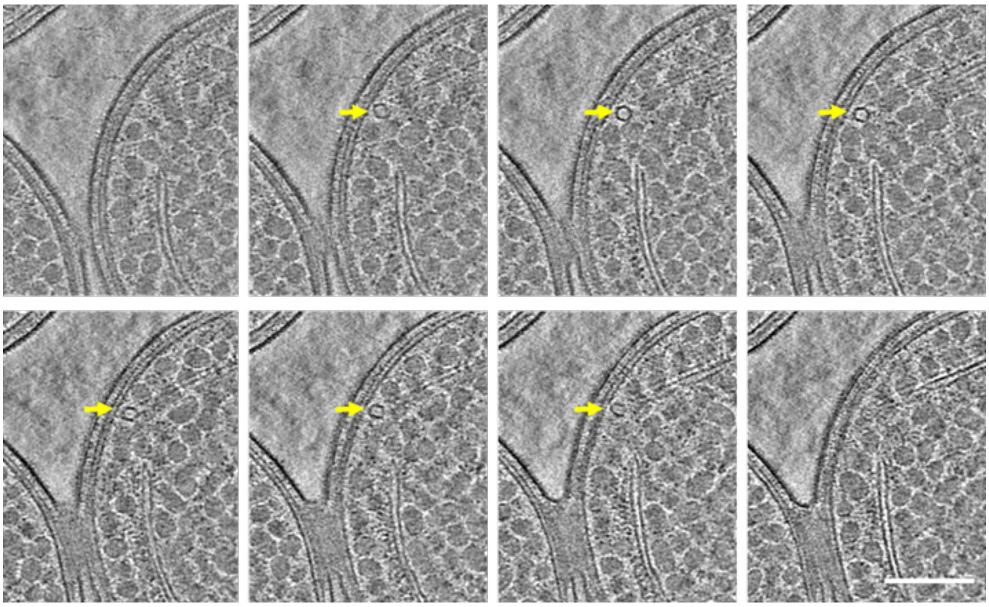

Supplementary Figure 7. Tomographic slices of a representative small tubular structure close to the thylakoid membrane in *Synechococcus*. Eight evenly spaced single slices (pixel size 2.49 nm), with the distance of 12.5 nm, are shown to enclose a tubular structure. Yellow arrows point to the tubular structure. Scale bar, 200nm. See Supplementary Movie 5. Experiment was repeated 3 times with similar results.

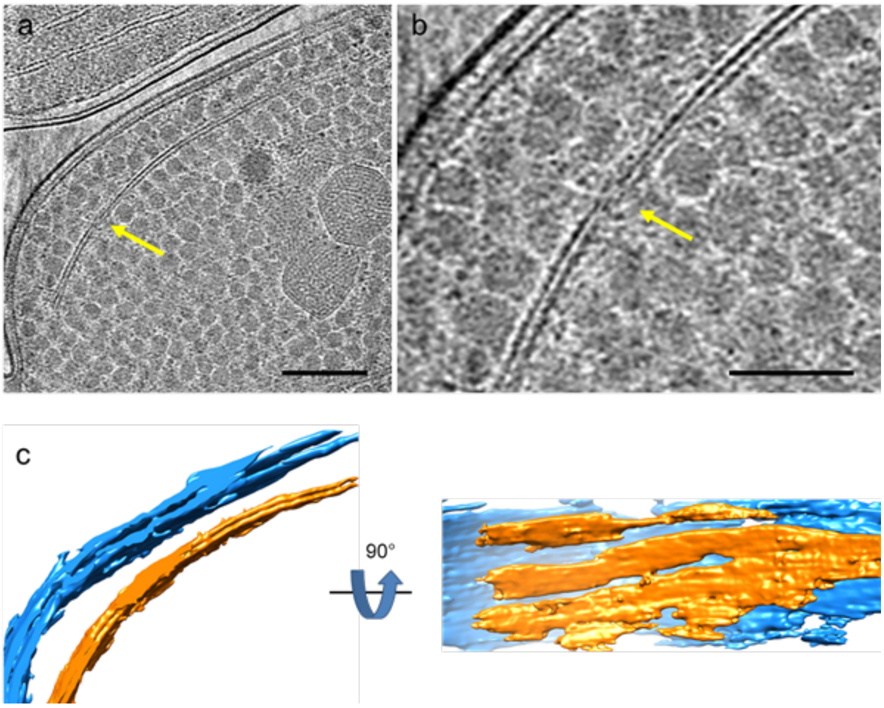

Supplementary Figure 8. Discontinued thylakoid membranes in *Synechococcus*. a-b, Tomographic slice of a *Synechococcus cell* (**a**) and its enlarged view (**b**), displaying perforations of thylakoid membranes (yellow arrows). Experiment was repeated 3 times with similar results. Slice thickness, 2.49 nm. Scale bars, 200 nm in **a** and 100 nm in **b**. **c,** Segmented volume of the tomogram shown in **a**. Outer and plasma membranes are presented in blue, thylakoid membranes in gold. See Supplementary Movie 6.

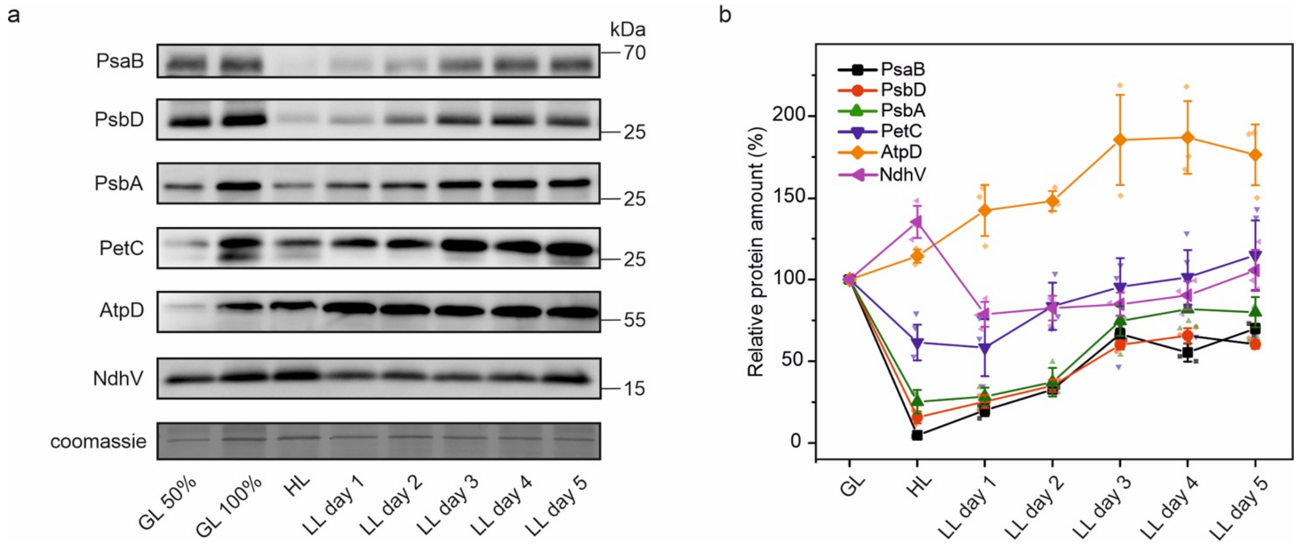

Supplementary Figure 9. Immunoblot analysis of photosynthetic thylakoid membrane during thylakoid biogenesis. Membrane protein fractions were isolated from *Synechococcus* cells grown under GL, HL, and HL-grown cells transferred to LL. a, Immunoblotting with protein-specific antibodies. 10–25 µg (100 %) of isolated membrane proteins were loaded in each lane (15 µg for the immunoblot analysis using α-PsaB, α-PsbD and α-PsbA, 25 µg for α-PetC, α-AtpD and α-NdhV). Coomassie staining is presented as a loading control. **b,** Relative quantification of protein amounts from immunoblots. Values are means ± SD; n = 3 biologically independent experiments.

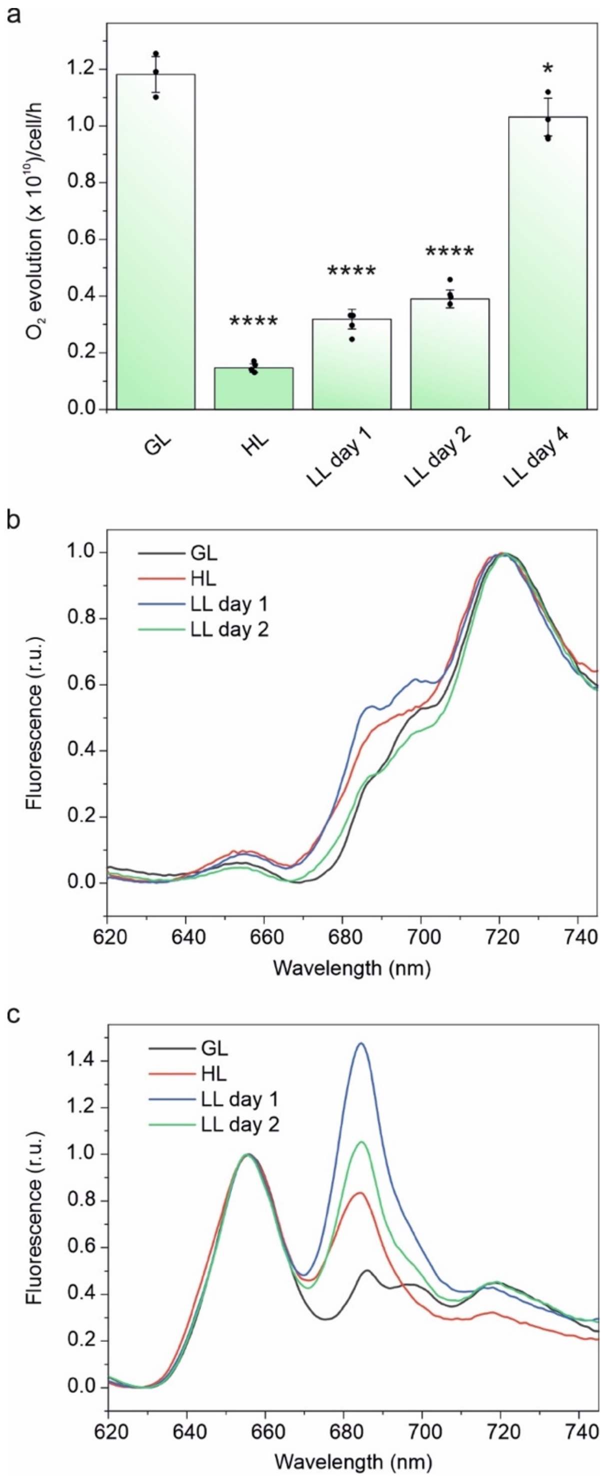

Supplementary Figure 10. Changes in the functional properties of photosynthetic machinery during light-regulated thylakoid membrane biogenesis in *Synechococcus*. Cells were grown under growth light (GL), high light (HL) and HL-grown cells were transferred to low light (LL) conditions for 2 to 4 days. **a,** The maximum capacity of PSII per cell determined with oxygen electrode in the presence of 2,5-Dichloro-1,4-benzoquinone (DCBQ) and ferricyanide, at saturating light intensity. Values are means ± SD; $n = 3$ biologically independent experiments for GL and $n = 4$ biologically independent experiments for HL, LL day 1, LL day 2 and LL day 4. Asterisks indicate the statistically significant difference compared to GL cells. For HL $p = 1.41 \times 10^{-6}$, for LL day 1 $p = 5.88 \times 10^{-6}$, for LL day 2 $p = 9.88 \times 10^{-6}$ and for LL day 4 $p = 3.53 \times 10^{-2}$. Statistical analysis was performed using two-sided two-sample t-Test. **b-c,** 77K fluorescence emission spectra when cells were excited with a 435 nm light and were normalized at 720 nm (**b**) or with a 600 nm light and were normalized at 655 nm (**c**). For GL curve is an average of 3biologically independent experiments and for HL, LL day 1 and LL day 2 curves are averages of 4 biologically independent experiments.

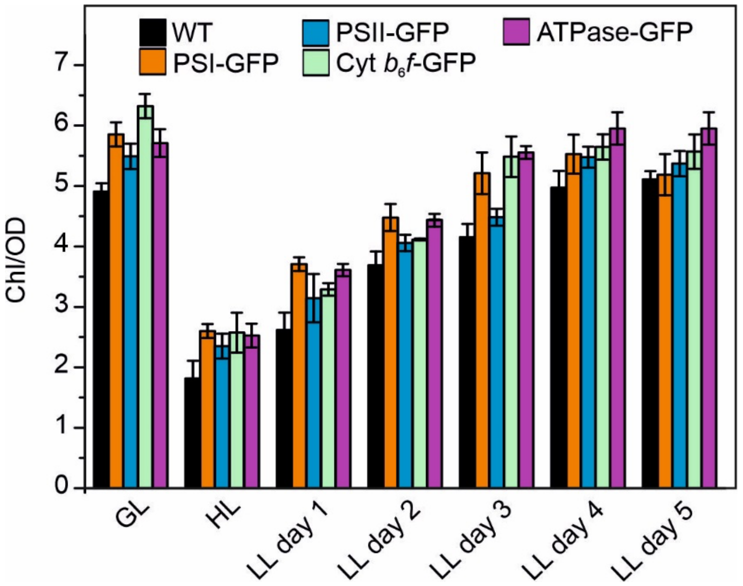

Supplementary Figure 11. The Chl amount (mg Chl per OD_{750}) of WT, PSI-, PSII-, Cyt *b_6f*-, and ATPase-GFP *Synechococcus* strains grown under GL, HL, and HL-grown cells transferred to LL. Values are means ± SD; *n* = 3 biologically independent experiments.

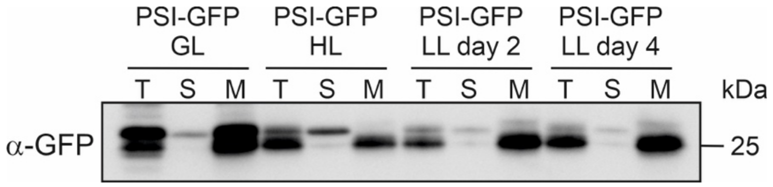

Supplementary Figure 12. Immunoblotting with GFP-specific antibody from the isolated total protein (T), the soluble (S) the membrane (M) fractions of the *Synechococcus* PSI-eGFP strain grown under GL, HL, and HL-grown cells transferred to LL for 2 and 4 days. 30 μg of proteins from each isolated fraction were loaded in each lane. Experiment was repeated 3 times with similar results.

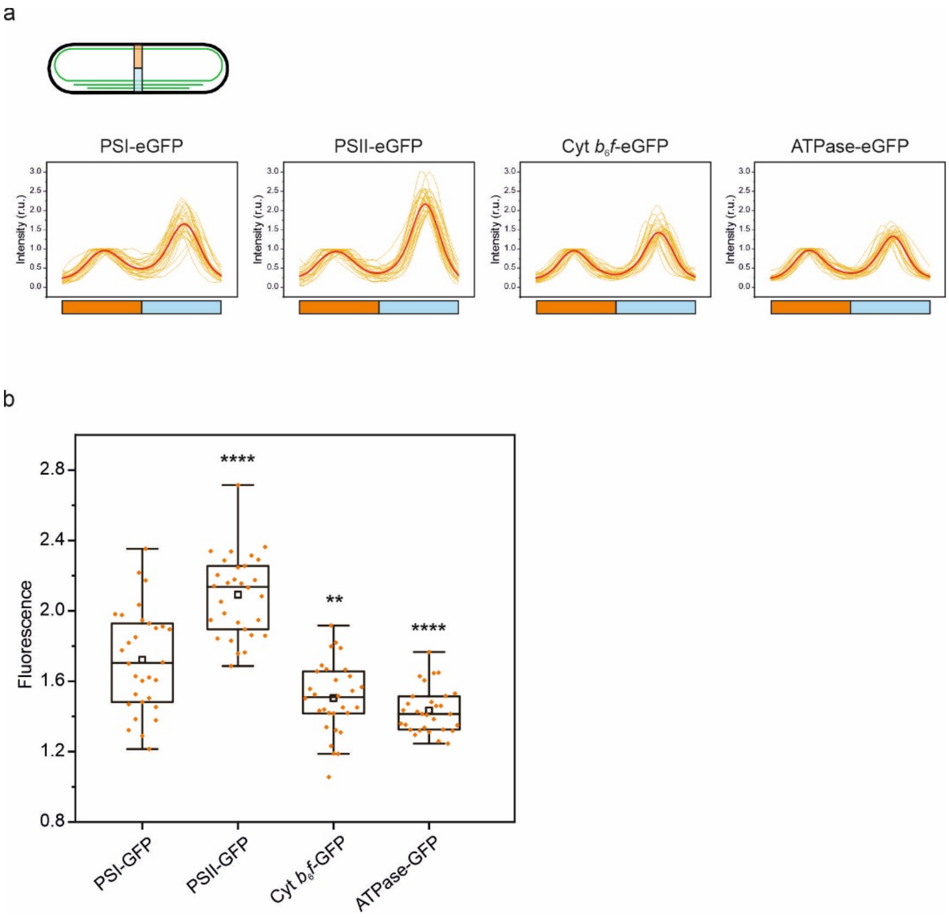

Supplemental Figure 13. Distribution of photosynthetic protein complexes between longitudinal cell sides at the beginning of thylakoid membrane biogenesis. a, Intensity of PSI-, PSII-, Cyt b_6f- and ATPase-eGFP signal across the cell in *Synechococcus* strains transferred from HL to LL conditions for one day. Profiles in red are averages from 30 individual cells (in yellow on the background) from 3 biologically independent experiments. The PSI-GFP, PSII-GFP, Cyt b_6f-GFP, and ATPase-GFP signals displayed a similar asymmetrical distribution as Chl autofluorescence signals. Orange bar in X-axis represents the longitudinal cell side with weaker Chl fluorescence representing fewer thylakoid layers (GFP fluorescence was normalized to 1), light blue bar with stronger Chl fluorescence representing more thylakoid layers. **b**, The unnormalized signal ratios of PSI-, PSII-, and Cyt b_6f-eGFP between longitudinal cell sides. n = 30 cells from three biologically independent experiments. The higher value corresponds to more uneven distribution between longitudinal cell sides. Box plots display the median (line), the average (open square), the interquartile range (box) and the whiskers (extending 1.5 times the interquartile range). Asterisks indicate the statistically significant differences compared to PSI-GFP signal. For PSII-GFP $p = 1.08 \times 10^{-6}$, for Cyt b_6f-eGFP $p = 1.37 \times 10^{-3}$, and for ATPase-GFP $p = 6.56 \times 10^{-6}$. Statistical analysis was performed using two-sided two-sample t-Test.

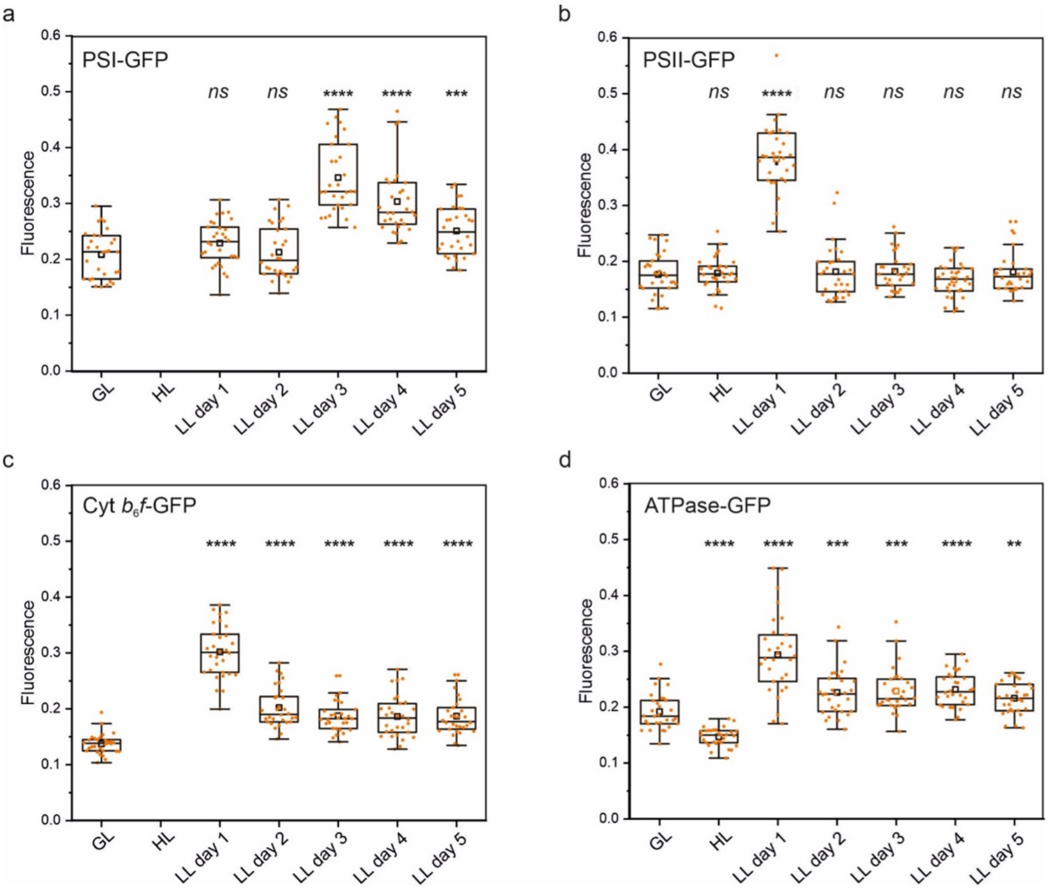

Supplementary Figure 14. Normalized deviation of eGFP-signal distribution along the thylakoid membranes in the *Synechococcus* strains grown under GL, HL, and HL-grown cells transferred to LL for 5 days. a, PSI-GFP, **b,** PSII-GFP, **c,** Cyt b_6f-GFP, and **d,** ATPase-GFP. $n = 30$ cells from 3 biologically independent experiments. The standard deviation of the signal normalized to the total fluorescence provides a quantitative measure of the patchiness of the signal. Evenly distributed fluorescence fluctuates little along the line profile and therefore has a low standard deviation, whereas the patchy distribution has a high standard deviation. Box plots display the median (line), the average (open square), the interquartile range (box) and the whiskers (extending 1.5 times the interquartile range). Asterisks indicate statistically significant difference to GL cells. For PSI-GFP LL3 $p = 3.37 \times 10^{-14}$, for PSI-GFP LL3 $p = 2.32 \times 10^{-9}$, for PSII-GFP LL 1 $p = 2.65 \times 10^{-22}$, for Cyt b_6f-GFP LL1 $p = 4.17 \times 10^{-25}$, for Cyt b_6f-GFP LL2 $p = 1.52 \times 10^{-12}$, for Cyt b_6f-GFP LL3 $p = 6.11 \times 10^{-11}$, for Cyt b_6f-GFP LL4 $p = 2.42 \times 10^{-8}$, for Cyt b_6f-GFP LL5 $p = 1.94 \times 10^{-9}$, for ATPase GFP HL $p = 5.97 \times 10^{-9}$, for ATPase GFP LL1 $p = 1.88 \times 10^{-9}$, for ATPase GFP LL2 $p = 7.22 \times 10^{-4}$, for ATPase GFP LL3 $p = 2.45 \times 10^{-4}$, for ATPase GFP LL4 $p = 8.97 \times 10^{-6}$ and for ATPase GFP LL5 $p = 3.35 \times 10^{-4}$. *ns*, not significant. Statistical analysis was performed using two-sided two-sample t-Test. Under HL PSI- and Cyt b_6f-GFP signals were not quantified due to unrestricted and weak signals, respectively.

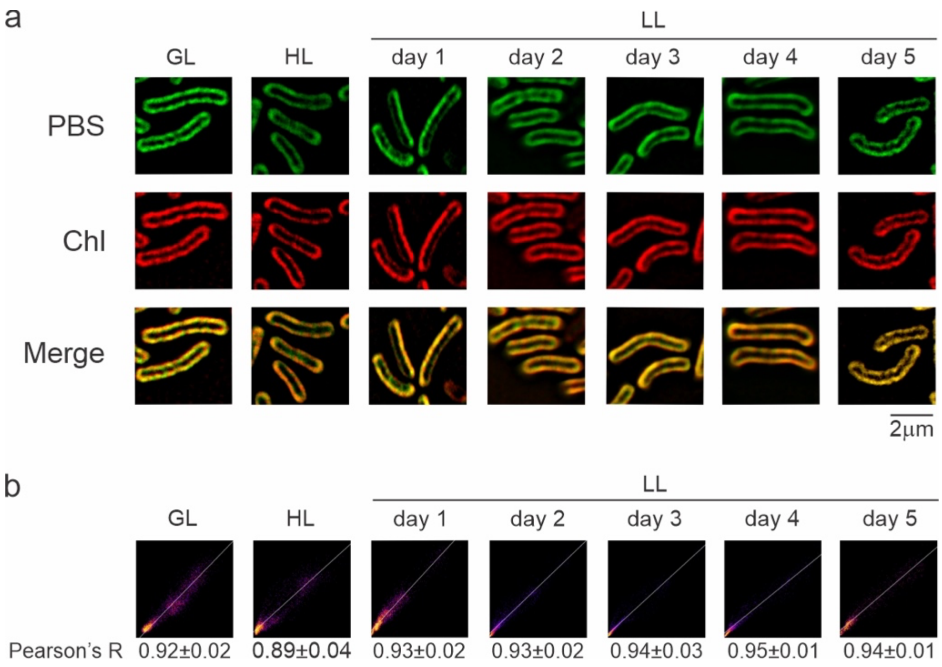

Supplementary Figure 15. Expression and intracellular localization of phycobilisomes (PBS) *in vivo* in *Synechococcus* during thylakoid membrane biogenesis. a, *Synechococcus* wild type (WT) cells imaged by Dragonfly spinning disk confocal microscope with the super-resolution radial fluctuations (SRRF)-stream technology. Figures are representative of 3 biologically independent experiments. First row: phycobilisome (PBS) fluorescence; second row: Chl autofluorescence; third row: merged channels. **b,** Scatter plots and Pearson's correlation values (± SD) for the colocalization of PBS and Chl for each studied condition. PBS and Chl fluorescence were analyzed from 30 individual cells in total from 3 biologically independent experiments.

Supplementary Table 1. Adjusted R^2 values and coefficient factors of the fourth-degree polynomial functions ($y = A_0 + A_1 \times x + A_2 \times x^2 + A_3 \times x^3 + A_4 \times x^4$) fitted for representative photosynthetic proteins from global quantification as a function of time.

		R^2	A_0	A_1	A_2	A_3	A_4
**PSI**	**PsaA**	0.98151	0.28678	-0.26066	0.15683	-0.02681	0.00156
	**PsaB**	0.99498	0.21329	-0.14085	0.10322	-0.01846	0.00119
	**PsaC**	0.99935	-0.00808	0.19384	-0.10782	0.03455	-0.00323
	**PsaL**	0.99967	0.17845	-0.12683	0.06925	-0.00497	-0.000211
**PSII**	**D1**	0.99936	0.47404	-0.4330	0.22175	-0.03398	0.00165
	**D2**	0.97432	0.18144	0.12393	-0.07865	0.03053	-0.00312
	**PsbF**	0.99486	-0.90941	2,10455	-0.59974	0.06652	-0.00258
	**CP 47**	0.96176	0.43509	-0.27907	0.11229	-0.00707	-0.00052
	**CP43**	0.99982	0.20142	-0.1377	0.07576	-0.00775	0.00008
	**PsbL**	0.97164	-0.39766	0.94209	-0.40186	0.08143	-0.00591
**Cyt b_6f**	**PetA**	0.99612	0.12287	0.00828	0.03599	0.00525	-0.00151
	**PetB**	0.99497	0.52441	-0.20539	0.06287	0.00293	-0.00115
	**PetC**	0.88053	0.49802	-0.07445	0.01305	0.01215	-0.00188
	**PetD**	0.91018	0.92673	-1.00638	0.52771	-0.09919	0.00633
**NDH-1**	**NdhD4**	0.94908	-0.0756	0.87649	-0.47535	0.10924	-0.0084
	**NdhF4**	0.88885	1.05128	-1.25608	0.75139	-0.15815	0.01097
	**NdhF3**	0.52203	0.92361	5.09378	-2.85121	0.56635	-0.03785
	**CupA**	0.97552	6.95398	-0.94335	-0.46855	0.16794	-0.01442
	**NdhE**	0.61526	4.06961	-2.8782	1.17481	-0.20318	0.01228
