# Supplementary material for: Probing the biogenesis pathway and dynamics of thylakoid membranes
Source: Nat Commun. 2021 Jun 9;12:3475. doi: 10.1038/s41467-021-23680-1 (PMC8190092; doi:10.1038/s41467-021-23680-1)
Supplement: Supplementary file 2 — Descriptions of Additional Supplementary Files [file 41467_2021_23680_MOESM2_ESM.pdf]

Descriptions of Additional Supplementary Files

Supplementary Movie 1
Description: Tomographic slice view of Synechococcus cell lamella with segmented volume accompanying Fig. 1a, 1b. Sequential sections through the tomographic volumes in orthographic view and corresponding segmentations, showing cell membrane (blue), thylakoid membranes (gold), glycogen granules (cyan), carboxysomes (green), large dense granules (pink) and tubular structures (red). Regions containing thylakoid membrane disconnections and thylakoid membrane branching are zoomed-in sequentially.

Supplementary Movie 2
Description: A close-up view of tomographic slices in Synechococcus, accompanying Fig. 2c, 2d.

Supplementary Movie 3
Description: A close-up view of the thylapse-like structure in Synechococcus, accompanying Fig. 1e, 1f. Sequential sections through the tomographic volumes in orthographic view and corresponding segmentations, showing cell membrane (blue), thylakoid membranes (gold), glycogen granules (cyan). Regions containing close contact between cyanobacterial inner membrane and thylakoid membrane are zoomed-in sequentially.

Supplementary Movies 4 and 5
Description: Close-up views of small segments (indicated by yellow arrow) of thylakoid membranes near thylakoid membrane breakages (indicated by black arrow) close to the plasma membrane in Synechococcus, accompanying Supplementary Fig. 5.

Supplementary Movie 6
Description: A close-up view of small, vesicle-like structure (indicated by yellow arrow) locating close to the plasma and thylakoid membrane in Synechococcus. The movie accompanies Supplementary Fig. 6, 7.

Supplementary Movie 7
Description: Tomographic slice view of Synechococcus cell lamella with segmented volume showing the perforations of thylakoid membranes, accompanying Supplementary Fig. 8. Sequential sections through the tomographic volumes in orthographic view and corresponding segmentations, showing cell membrane (blue), thylakoid membranes (gold), glycogen granules (cyan), carboxysomes (green), and large dense granules (pink). Regions containing thylakoid membrane disconnections are zoomed-in sequentially.

Supplementary Data 1
Description: Global protein quantification of Synechococcus grown under growth light (GL), high light (HL) and HL-grown cells transferred to low light (LL) conditions for 5 days. Data are from 3 biologically independent experiments.
