# Supplementary material for: Probing the biogenesis pathway and dynamics of thylakoid membranes
Source: Nat Commun. 2021 Jun 9;12:3475. doi: 10.1038/s41467-021-23680-1 (PMC8190092; doi:10.1038/s41467-021-23680-1)
Supplement: Supplementary file 11 — Reporting Summary [file 41467_2021_23680_MOESM11_ESM.pdf]

nature research

Corresponding author(s): Professor Luning Liu

Last updated by author(s): May 4, 2021

Reporting Summary

Nature Research wishes to improve the reproducibility of the work that we publish. This form provides structure for consistency and transparency in reporting. For further information on Nature Research policies, see our Editorial Policies and the Editorial Policy Checklist.

Statistics

For all statistical analyses, confirm that the following items are present in the figure legend, table legend, main text, or Methods section.

n/a	Confirmed	
☐	☒	The exact sample size (*n*) for each experimental group/condition, given as a discrete number and unit of measurement
☐	☒	A statement on whether measurements were taken from distinct samples or whether the same sample was measured repeatedly
☐	☒	The statistical test(s) used AND whether they are one- or two-sided<br>*Only common tests should be described solely by name; describe more complex techniques in the Methods section.*
☒	☐	A description of all covariates tested
☐	☒	A description of any assumptions or corrections, such as tests of normality and adjustment for multiple comparisons
☐	☒	A full description of the statistical parameters including central tendency (e.g. means) or other basic estimates (e.g. regression coefficient) AND variation (e.g. standard deviation) or associated estimates of uncertainty (e.g. confidence intervals)
☐	☒	For null hypothesis testing, the test statistic (e.g. *F*, *t*, *r*) with confidence intervals, effect sizes, degrees of freedom and *P* value noted<br>*Give P values as exact values whenever suitable.*
☒	☐	For Bayesian analysis, information on the choice of priors and Markov chain Monte Carlo settings
☒	☐	For hierarchical and complex designs, identification of the appropriate level for tests and full reporting of outcomes
☒	☐	Estimates of effect sizes (e.g. Cohen's *d*, Pearson's *r*), indicating how they were calculated

Our web collection on statistics for biologists contains articles on many of the points above.

Software and code

Policy information about availability of computer code

Data collection	Transmission electron microscopy (TEM) images were recorded using an FEI Tecnai G2 Spirit BioTWIN transmission electron microscope equipped with a Gatan Rio 16 camera.<br>Immunoblot images were collected by ImageQuant LAS 4000 software version 1.2.1.119.<br>Data-dependent LC-MS/MS analysis was conducted on a QExactive quadrupole-Orbitrap mass spectrometer coupled to a Dionex Ultimate 3000 RSLC nano-liquid chromatograph (Hemel Hempstead, UK). The raw data file was imported into Progenesis QI for Proteomics (Version 3.0 Nonlinear Dynamics, Newcastle upon Tyne, UK, Waters Company).<br>For cryo-electron tomography (ET) tilt-series were acquired from lamella using a Titan Krios microscope (Thermo Fisher Scientific) operated at 300 kV. Micrographs were recorded with a K3 camera equipped with a Gatan Quantum energy filter operated in zero-loss mode with a 30 eV slit width.<br>Live-cell super-resolution spinning disc confocal fluorescence imaging was performed on a Dragonfly microscope (Andor) utilizing super-resolution radial fluctuations (SRRF)-Stream technology controlled by Phusion 2.0 software.<br>Whole-cell absorption spectra were measured at room temperature using a modernised Aminco DW2000 UV/Vis spectrophotometer (Olis, USA).<br>Oxygen evolution was measured at 20°C in a Clarke-type oxygen electrode (Hansatech, UK) controlled by OxyLab2 software.<br>The 77K fluorescence emission spectra were measured using a Perkin-Elmer LS50 luminescence spectrometer (Foster City, CA) controlled by FL WinLab version 4.00.02 software.
Data analysis	TEM, confocal microscopy and immunoblot data were analyzed with ImageJ software version 1.52h.<br>Concerning Cryo-EM data movie frames were motion-corrected using MotionCor2. Tilt-series alignment was performed using IMOD. Tomogram was reconstructed using 4 × binned (24.92 Å/pixel) micrographs without CTF correction with 5 cycles of SIRT. The automated segmentation/annotation of 3D volumes was performed using neuronal network implemented in EMAN2.3, with additional manual

adjustements. These volumes were visualized in 3D using UCSF Chimera.
Raw data files were searched against the UniProt proteomes database of Synechococcus elongatus 7942 (UniProt ID: UP000002717) using Proteome Discoverer software (Thermo Fisher Scientific version 1.4.1.14) connected to an in-house Mascot server (Matrix Science, version 2.4.1).Raw mass spectral data files were processed using Progenesis-QI (v4.1; Nonlinear Dynamics) to determine total protein abundances. Statistical analysis and graphs were performed using OriginPro 8.5 and Microsoft Excel 2016.

For manuscripts utilizing custom algorithms or software that are central to the research but not yet described in published literature, software must be made available to editors and reviewers. We strongly encourage code deposition in a community repository (e.g. GitHub). See the Nature Research guidelines for submitting code & software for further information.

Data

Policy information about availability of data

All manuscripts must include a data availability statement. This statement should provide the following information, where applicable:
- Accession codes, unique identifiers, or web links for publicly available datasets
- A list of figures that have associated raw data
- A description of any restrictions on data availability

Mass spectrometry proteomics data were deposited to the ProteomXhange Consortium via PRIDE partner repository with the Project accession PXD019731 (https://www.ebi.ac.uk/pride/archive/projects/PXD019731).

Fig. 1b, 1c, 3b-3e, 4, 5b, 5d, 5f, and 5h addition to Supplementary Fig. 1b, 1c, 6b, 6c, 9, 10, 11, 12, 13, 14, and 15 have associated raw data which are provided in Source Data file.

Field-specific reporting

Please select the one below that is the best fit for your research. If you are not sure, read the appropriate sections before making your selection.

☒ Life sciences ☐ Behavioural & social sciences ☐ Ecological, evolutionary & environmental sciences

For a reference copy of the document with all sections, see nature.com/documents/nr-reporting-summary-flat.pdf

Life sciences study design

All studies must disclose on these points even when the disclosure is negative.

Sample size	At least three independent biological cultures from each experiment condition were used for TEM and confocal imaging. Under each condition, high quality images were obtained with good biological reproducibility. The sample size for SDS-PAGE, Blue-Native-PAGE, immunoblotting, mass spectrometry, absorption spectra, 77K fluorescence emission spectra, oxygen evolution, Chl determination and cell counting was not predetermined using statistical methods. At least three biological independent samples were purified and analyzed by SDS-PAGE, Blue-Native-PAGE, immunoblotting, mass spectrometry, absorption spectra, 77K fluorescence emission spectra, oxygen evolution, Chl determination and cell counting. Similar results were obtained from the replicates of each experimental analysis.
Data exclusions	Representative high quality TEM and confocal images were shown in the paper. Low quality and low resolution images were excluded, which is standard practice for TEM and confocal imaging and can be considered as pre-established criteria.
Replication	All of the experiments were repeated more than three times, and were reproduced successfully.
Randomization	Cells were selected randomly for imaging and biophysical measurements. The samples for electrophoresis and Western blot assay were mixed evenly.
Blinding	Blinding was not possible because sample preparation and data collection were conducted by the same investigators.

Reporting for specific materials, systems and methods

We require information from authors about some types of materials, experimental systems and methods used in many studies. Here, indicate whether each material, system or method listed is relevant to your study. If you are not sure if a list item applies to your research, read the appropriate section before selecting a response.

Materials & experimental systems

n/a	Involved in the study
☐	☒ Antibodies
☒	☐ Eukaryotic cell lines
☒	☐ Palaeontology and archaeology
☒	☐ Animals and other organisms
☒	☐ Human research participants
☒	☐ Clinical data
☒	☐ Dual use research of concern

Methods

n/a	Involved in the study
☒	☐ ChIP-seq
☒	☐ Flow cytometry
☒	☐ MRI-based neuroimaging

Antibodies

Antibodies used

Anti-PsaB antibody: specific to PsaB protein; Supplier name: Agrisera; Catalog Number: AS10 695; Clone name: unknown; Lot number: unknown; Dilution: 1:1000.
Anti-PsbA antibody: specific to PsbA protein (D1, DE-loop) ; Supplier name: Agrisera; Catalog Number: AS10 704 ;Clone name: unknown; Lot number: unknown; Dilution: 1:4000.
Anti-PsbD antibody: specific to PsbD protein (D2); Supplier name: Agrisera; Catalog Number: AS06 146 ;Clone name: unknown; Lot number: unknown; Dilution: 1:4000.
Anti-PetC antibody: specific to Rieske iron-sulfur protein of Cyt b6/f complex; Supplier name: Agrisera; Catalog Number: AS08 330; Clone name: unknown; Lot number: unknown; Dilution: 1:1500.
Anti-AtpB antibody: specific to AtpB protein; Supplier name: Agrisera; Catalog Number: AS05 085; Clone name: unknown; Lot number: unknown; Dilution: 1:3000.
Anti-NdhV antibody: (customization); Supplier name: gift from Dr. Hualing Mi, Dilution 1:4000
Anti-GFP antibody: specific to GFP protein; Supplier name: ; Catalog Number: A-11122 ; Clone name: unknown; Lot number: unknown; Dilution: 1:5000.
Goat anti-Mouse IgG, HRP Conjugate; Supplier name: Agrisera; Catalog number: AS111772; Clone name: unknown; Lot number: unknown; Dilution: 1:10000.
Goat anti-Rabbit IgG, HRP Conjugate; Supplier name: Agrisera; Catalog number: AS09602; Clone name: unknown; Lot number: unknown; Dilution: 1:10000.

Validation

Anti-PsaB antibody: confirmed reactivity with Arabidopsis thaliana, Brassica napus, Brassica rapa, Bryopsis corticulans, Echinola crus-galli, Hordeum vulgare, Neochloris oleoabundans (chlorophyta), Nicotiana tabacum, Pisum sativum, Solanum lycopersicum, Synechococcus sp. PCC7942, Synechocystis sp. PCC 6803, Triticum aestivum, Zea mays. Relevant citations: Lima-Melo et al. (2019). Plant J. doi: 10.1111/tpj.14177; Frede et al. (2019). J Photochem Photobiol B. 193:18-30. doi: 10.1016/j.jphotobiol. 2019.02.001.
Anti-PsbA: confirmed reactivity with: A.thaliana, C.reinhardii, H.vulgare, M.truncatula N. oleoabundans UTEX 1185 (chlorophyta), P.patens, P.sativum, S.vulgaris, S.alba, S.oleracea, Synechococcus sp. PCC 7942, Synechocystis sp. PCC6803, Triticum sp. Relevant Citations: Grieco et al. (2020). Adjustment of photosynthetic activity to drought and fluctuating light in wheat. Plant Cell Environ. 2020 Mar 16. doi: 10.1111/pce.13756; Rantala et al. (2020). PGR5 and NDH-1 systems do not function as protective electron acceptors but mitigate the consequences of PSI inhibition. Biochim Biophys Acta Bioenerg. 2020 Jan 11;1861(3):148154. doi: 10.1016/j.bbabio.2020.148154.
Anti-PsbD: confirmed reactivity with A. thaliana, Anabaena 7120, D. brightwellii, H. vulgare, C.reinhardtii, C. zofingiensis, L. corniculatus, N. tabacum, O. sativa, P. sativum, P. vulgaris, P. tricornutum, T. pratense, S. alba, Synechococcus sp. PCC 7942, Synechocystis sp. PCC6803, T. guillardii, T. pseudonana, Triticale, U. prolifera, Z. mays. Relevant citations: Amstutz et al. (2020). An atypical short-chain dehydrogenaseÃ¢??reductase functions in the relaxation of photoprotective qH in Arabidopsis. Nat Plants , 6 (2), 154-166; Lv et al. (2019). Uncoupled Expression of Nuclear and Plastid Photosynthesis-Associated Genes Contributes to Cell Death in a Lesion Mimic Mutant. Plant Cell. 2019 Jan;31(1):210-230. doi: 10.1105/tpc.18.00813.
Anti-PetC: Confirmed reactivity: A. thaliana, B. rapa subsp. chinensis, C. reinhardtii, E. crus-galli, Euglena sp., H. pluvialis, N. tabacum, P. miliaceum, P. sativum, S. oleracea, Synechococcus PCC 7942, Synechocystis sp. PCC 6803, Thalassiosira guillardii, Z. mays. Relevant citations: Zhang et al. (2020). Enhanced Relative Electron Transport Rate Contributes To Increased Photosynthetic Capacity In Autotetraploid Pak Choi. Plant Cell Physiol. 2020 Jan 6. pii: pcz238. doi: 10.1093/pcp/pcz238.; Du et al. (2018). Galactoglycerolipid Lipase PGD1 Is Involved in Thylakoid Membrane Remodeling in Response to Adverse Environmental Conditions in Chlamydomonas. Plant Cell. 2018 Feb;30(2):447-465. doi: 10.1105/tpc.17.00446.
Anti-AtpB antibody: the anti-AtpB antibody will detect the mitochondrial form of the F1 ATP synthase subcomplex, as well as the chloroplastic CF1 Atp Synthase, and most known bacterial F-type Atp Synthases. Peptide used for antibody production is located in a beta sheet, which is partly exposed near the surface of the AtpB protein. Anti-AtpB antibody was used as a loading control in Chlamydomonas reinhardtii and Synechocystis sp. PCC6803. Relevant citations: Aihara et al. (2019). Nat Plants 5(1):34-40. doi: 10.1038/s41477-018-0332-5; Gabilly et al. (2019). Proc Natl Acad Sci U S A. pii: 201821689. doi: 10.1073/pnas.1821689116.
Anti-NdhV antibody: proven to specific in Chen, X., He, Z., Xu, M. et al. NdhV subunit regulates the activity of type-1 NAD(P)H dehydrogenase under high light conditions in cyanobacterium Synechocystis sp. PCC 6803. Sci Rep 6, 28361 (2016). https://doi.org/10.1038/srep28361.
Anti-GFP: Polyclonal antibody, validation statement on manufacturer's website https://www.thermofisher.com/antibody/product/GFP-Antibody-Polyclonal/A-11122
